# Disruption of RCAN1.4 expression mediated by YY1/HDAC2 modulates chronic renal allograft interstitial fibrosis

**DOI:** 10.1038/s41420-023-01574-z

**Published:** 2023-07-28

**Authors:** Jianjian Zhang, Yao Zhang, Dengyuan Feng, Hai Zhou, Zeping Gui, Ming Zheng, Zhou Hang, Min Gu, Ruoyun Tan

**Affiliations:** Department of Urology, the First Affiliated Hospital of Nanjing Medical University, Jiangsu Province Hospital, 210029 Nanjing, China

**Keywords:** Stress signalling, End-stage renal disease, Transcriptional regulatory elements

## Abstract

Chronic allograft dysfunction (CAD) is a major factor that hinders kidney transplant survival in the long run. Epithelial–mesenchymal transition (EMT) has been confirmed to significantly contribute to interstitial fibrosis/tubular atrophy (IF/TA), which is the main histopathological feature of CAD. Aberrant expression of the regulator of calcineurin 1 (RCAN1), recognized as an endogenous inhibitor of the calcineurin phosphatase, has been shown to be extensively involved in various kidney diseases. However, it remains unclear how RCAN1.4 regulates IF/TA formation in CAD patients. Herein, an in vivo mouse renal transplantation model and an in vitro model of human renal tubular epithelial cells (HK-2) treated with tumor necrosis factor-α (TNF-α) were employed. Our results proved that RCAN1.4 expression was decreased in vivo and in vitro, in addition to the up-regulation of Yin Yang 1 (YY1), a transcription factor that has been reported to convey multiple functions in chronic kidney disease (CKD). Knocking in of RCAN1.4 efficiently attenuated chronic renal allograft interstitial fibrosis in vivo and inhibited TNF-α-induced EMT in vitro through regulating anti-oxidative stress and the calcineurin/nuclear factor of activated T cells cytoplasmic 1 (NFATc1) signaling pathway. In addition, suppression of YY1 mediated by shRNA or siRNA alleviated TNF-α-induced EMT through abolishing reactive species partly in an RCAN1.4-dependent manner. Notably, we confirmed that YY1 negatively regulated RCAN1.4 transcription by directly interacting with the RCAN1.4 promoter. In addition, histone deacetylase 2 (HDAC2) interacted with YY1 to form a multi-molecular complex, which was involved in TNF-α-induced RCAN1.4 transcriptional repression. Therefore, RCAN1.4 is suggested to be modulated by the YY1/HDAC2 transcription repressor complex in an epigenetic manner, which is a mediated nephroprotective effect partly through modulating O2⋅− generation and the calcineurin/NFATc1 signaling pathway. Thus, the YY1–RCAN1.4 axis constitutes an innovative target for IF/TA treatment in CAD patients.

## Introduction

Kidney transplantation is the optimum course of action for patients with uremia to improve their quality of life [1]. There are many factors leading to CAD in renal transplant recipients [2]. Interstitial fibrosis/tubular atrophy (IF/TA) of transplanted kidneys, one of the primary pathological features of CAD, is a leading cause affecting the long-term survival of renal allograft [3, 4]. It is well-accepted that excessive extracellular matrix (ECM), mainly produced by myofibroblasts, is abnormally deposited in the renal interstitium, subsequently leading to IF/TA in compromised kidneys [5]. Multiple studies have demonstrated that many factors, including vasoconstriction, oxidative stress, fibroblast activation, epithelial–mesenchymal transition (EMT), endothelial–mesenchymal transition (EndMT), etc., are involved in the progress of ECM deposition and IF/TA [6–8]. However, the key mechanism of IF/TA formation in CAD patients has not been completely clear yet.

EMT refers to the biological process by which epithelial cells are converted into cells with an interstitial phenotype in certain pathological conditions [9]. Current studies have shown that EMT participated in embryogenesis, malignancy metastasis, and organ fibrosis (including lung, kidney, and liver) [10, 11]. In the process of IF/TA in transplanted kidneys, allografts are reported to be attacked by several serum inflammatory cytokines-mediated immune responses [7]. Moreover, during EMT in renal allografts, renal tubular epithelial cells are attacked by several inflammatory cytokines, among which TNF-α was considered the most important [12, 13]. In our previous studies, TNF-α was proved to be a crucial factor in regulating the development of EMT in renal allografts fibrosis [14, 15]. In particular, inflammatory cytokines accumulation leads to excessive reactive oxygen species (ROS), such as O2⋅- and ⋅OH ultimately contributes to IF/TA formation [12, 16]. Some preclinical studies have shown that biopsy samples with CAD experience increased oxidative stress [17]. During the chronic IF/TA of renal allograft pathogenesis, the accumulation of ROS triggers the transdifferentiation of tubular epithelial cells [18, 19]. In a study of the rat model of a kidney transplant, fenofibrate alleviated IF/TA in the renal allograft by inhibiting oxidative stress-induced EMT [20].

RCAN1, also referred to as DSCR1/MCIP1, composed of four different transcripts (RCAN1.1S, RCAN1.1SL, RCAN1.2, and RCAN1.4) [21]. The family of RCAN1, especially RCAN1.4, has been shown extensively involved in various physiopathological processes, including regulation of ROS production, mitochondrial dynamics, thermogenesis, etc. [22]. A study has shown that lacking RCAN1, ROS levels increase, indicating that RCAN1 has antioxidant effects to some extent [23]. Our previous study showed that RCAN1.4 attenuated UUO-induced renal fibrosis through inhibiting calcineurin/NFAT2 pathway [24]. However, the precise mechanisms of RCAN1.4 in renal allografts with IF/TA remain unclear. It is widely recognized that the epigenetic mechanism produces a marked effect by interacting with DNA, mRNA, or histone to modify gene expression. It has demonstrated that methylation of RCAN1.4 mediated by DNMT1 and DNMT3 aggerated liver fibrosis [25]. YY1, predicted to interact with the RCAN1.4 promoter, is a widely expressed multifunctional zinc finger DNA binding transcription factor in cells [26]. Furthermore, YY1 has been reported to convey multiple functions in chronic kidney disease (CKD) [27]. Hence, we hypothesis there is a novel mechanism in which YY1-mediated RCAN1.4 expression in epigenetic aspect in CAD patients.

In this research, we explored how RCAN1.4 modulates IF/TA in the renal allograft of CAD and explored whether the expression of RCAN1.4 in CAD was attributed to transcriptional regulation of YY1, providing a theoretical basis for treating renal interstitial fibrosis in CAD patients.

## Results

### RCAN1.4 expression was down-regulated in vivo and in vitro

**Fig. 1:**
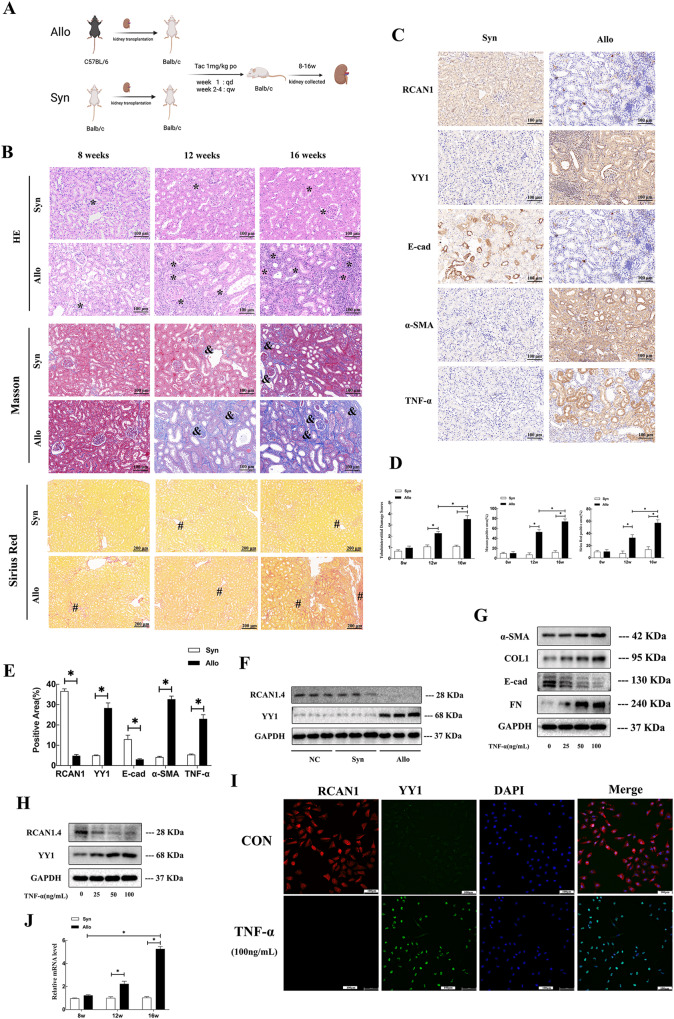
RCAN1.4 expression was down-regulated in vivo and in vitro. The mouse renal transplanted models of chronic renal allograft interstitial fibrosis were established as described in methods and materials. **A** Schematic diagram of mouse experimental process. **B** HE, Masson trichrome staining and Sirius Red staining stains of kidney interstitium tissue of the two groups of mice (×100, scale bar, 100 μm). Renal parenchyma exhibiting tubular dilation (black *) and ECM deposition in interstitial space of kidney (black # and &). **C** Immunohistochemistry signals of RCAN1, YY1, E-cad, α-SMA, and TNF-α. **D** Tubulointerstitial damage scores and the proportion of Masson’s positive area were assessed in different groups (*n* = 6, *P < 0.05). **E** Statistical analysis of the protein’s positive area was performed in different groups (n = 6, *P < 0.05). **F** Western blotting analysis was performed after collecting kidney lysates. Representative blots of RCAN1.4 and YY1(*n* = 6). In vivo, TNF-α(0–100 ng/mL) was applied to HK‐2 cells to induce EMT. (**G**-**H**) Representative blots of RCAN1.4, YY1, EMT markers (COL1, FN, E-cad and α-SMA) (*n* = 3). **I** Co-localization of RCAN1 and YY1 protein expression in TNF-α-stimulated HK-2 cells was examined by immunofluorescence (scale bars, 100 μm). **J** The mRNA levels of TNF-α were detected by qPCR (*n* = 3). The results are expressed as the mean ± standard error of the mean (SEM). for 3–4 independent experiments. ^*^p < 0.05, as indicated.

To determine the possible link between the transcription factor YY1 and RCAN1.4 in renal allografts, we first established a mouse renal transplant model of chronic renal allograft IF by kidney transplantation from C57BL/6 mice to BALB/c mouse (Allo group). H&E, Masson staining, and Sirius Red staining were performed to validate the severity of renal allograft fibrosis. Compared with the Syn group, renal fibrosis with inflammatory cell infiltration, tubular atrophy, and renal interstitial fibrosis, including indicating ECM deposition, were observed in mouse allografts taken at 12 and 16 weeks after kidney transplantation in the Allo group (Fig. 1A–E). In addition, a rapid accumulation of serum BUN and Cr was found in the Allo group compared with the Syn group, which was further augmented with increasing time of chronic renal allograft IF (Supplementary Fig. S1A, B). To detect the progression of EMT in the allograft kidneys, Western blot assays, and immunohistochemical staining were performed. The results indicated high expression of fibronectin (FN), collagen type 1 (COL1), and alpha-smooth muscle actin (α-SMA), and reduced expression of E-cadherin (E-cad), in the Allo group compared with the Syn group (Supplementary Fig. S1C). In parallel, the immunostaining results corresponded with the Western blot assays (Fig. 1E). Moreover, the protein level of RCAN1.4 was dominantly reduced while the expression of YY1 became elevated quickly in the Allo group compared with the Syn group (Fig. 1F). The immunohistochemistry results also confirmed a similar trend of RCAN1.4 and YY1 expression to that of the Western blot assays (Fig. 1C, E). Furthermore, A large portion of the YY1 expression was found in the nucleus (Fig. 1C). Besides, the expression of TNF-α was elevated quickly in the Allo group compared with the Syn group (Fig. 1C, J). Since excessive oxidative stress leads to renal IF, after kidney transplantation, we then assessed the accumulation of ROS, such as O2⋅−, in mice. Western blotting analysis revealed a decreased expression of antioxidant genes, including catalase, superoxide dismutase 1 (SOD1), and SOD2 in the Allo group (Supplementary Fig. S1D). In parallel, O2⋅− accumulation, detected by the HE probe, was observed in the Allo group (Supplementary Fig. S1E).

To further evaluate RCAN1.4 and YY1 expression in tubular epithelial cells undergoing pathological EMT, TNF-α (0–100 ng/mL) was administered to HK‐2 cells for 48 h. As shown in Fig. 1G, a decrease in E-cad expression and enhanced expression of FN, COL1, and α-SMA was observed in HK-2 cells following treatment with TNF-α. Furthermore, the expression level of EMT markers corresponded with the concentration of TNF-α. Importantly, in correspondence with the expression of YY1 and RCAN1.4 in vivo, the levels of YY1 mRNA and protein were up-regulated, which peaked at the 100 ng/mL concentration of TNF-α (Fig. 1H, Supplementary Fig. S1F). In parallel, the levels of RCAN1.4 mRNA and protein quickly declined in TNF-α-treated HK-2 cells, which were further augmented at 50 and 100 ng/mL TNF-α (Fig. 1F, Supplementary Fig. S1H). In addition, co-localization of RCAN1.4 and YY1 in TNF-α-stimulated HK-2 cells, as assessed by immunofluorescence, showed that YY1 was mainly located in the nucleus, while RCAN1.4 was in the cytoplasm (Fig. 1I). Collectively, we showed an association between the expression of RCAN1.4 and YY1 in renal allografts and in cultured HK-2 cells following TNF-α stimulation, unveiling a possible relationship between RCAN1.4 and YY1 in renal allograft IF.

### RCAN1.4-mediated suppression of TNF-α- induced EMT through the modulation of ROS in vitro

**Fig. 2:**
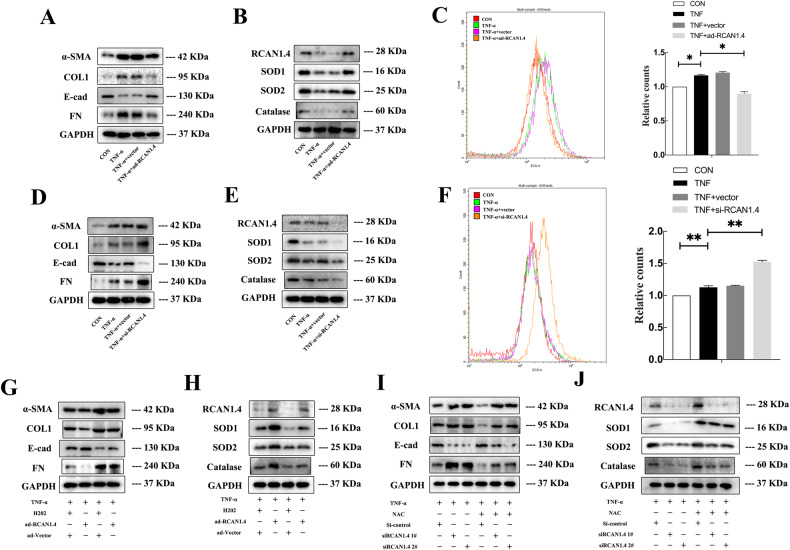
RCAN1.4-mediated suppressive function on EMT induced by TNF-α through modulating reactive species in vitro. HK-2 cells were transfected with RV230-RCAN1.4 plasmid or RCAN1.4-RNAi to change the expression of RCAN1.4, and treated with H_2_O_2_ or NAC with indicated concentration, followed by TNF-α stimuli. **A**, **D**, **G**, **I** Representative blots of EMT markers (COL1, FN, E-cad, and α-SMA) (*n* = 3). **B**, **E**, **H**, **J** Representative blots of anti-oxidant proteins (SOD1, SOD2, and Catalase) (*n* = 3). **C**, **F** O_2_⋅− levels were detected by HE probe and were quantified(*n* = 3). The results are expressed as the mean ± standard error of the mean (SEM). for 3–4 independent experiments. *p < 0.05, **p < 0.05, as indicated.

Reaffirming the importance of RCAN1.4 in regulating TNF-α-induced EMT in vitro, the RV230-RCAN1.4 plasmid and RCAN1.4-RNAi were employed to alter the expression of RCAN1.4 in HK-2 cells. Firstly, the expression of RCAN1.4 was remarkably up-regulated following transfection with the RV230-RCAN1.4 plasmid in HK-2 cells, subsequently reversing the protein expression of FN, COL1, α-SMA, and E-cad in HK-2 cells stimulated by TNF-α (Fig. 2A). Since the excessive accumulation of ROS, including O2⋅-, and a marked down-regulation of antioxidant genes, including catalase, SOD1, and SOD2, were observed on TNF-α-induced EMT in HK-2 cells (Supplementary Fig. S2A–C), we investigated the effect of RCAN1.4 overexpression on oxidative stress levels. As shown in Fig. 2B, the down-regulation of catalase, SOD1, and SOD2 induced by TNF-α was reversed by RCAN1.4 overexpression. In parallel, O2⋅− production triggered by TNF-α stimulation was abolished by overexpressing RCAN1.4 (Fig. 2C). In contrast, silencing of RCAN1.4 further promoted the expression of EMT markers and triggered oxidative stress induced by TNF-α (Fig. 2D–F). To further substantiate these observations, a ROS inhibitor (NAC) and inducer (H_2_O_2_) were used. Of note, overexpression of RCAN1.4 inhibited TNF-α-induced EMT in HK-2 cells, which was abolished by H_2_O_2_ stimulation (Fig. 2G, H). In contrast, the effect of silencing RCAN1.4, which aggravated EMT after TNF-α stimulation, was reversed by the ROS inhibitor NAC (Fig. 2I, J). All these studies suggest that RCAN1.4 can regulate EMT induced by TNF-α by affecting oxidative stress levels.

### rAAV-mediated overexpression of RCAN1.4 attenuated renal allograft IF

**Fig. 3:**
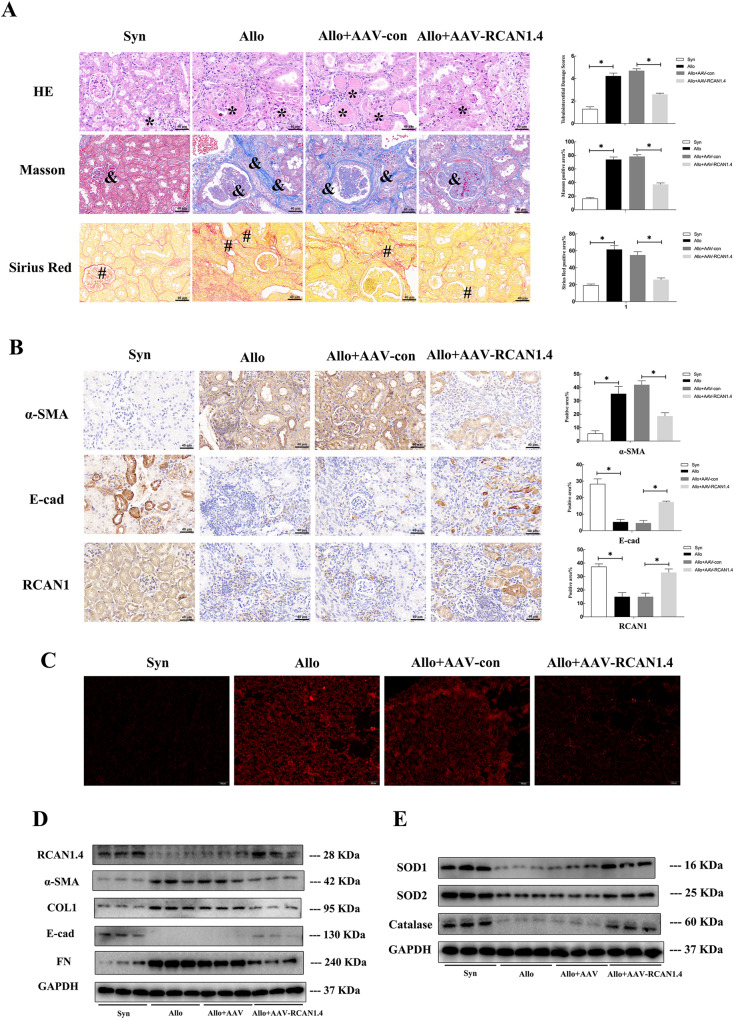
Recombinant adeno-associated virus-mediated overexpression of RCAN1.4 attenuated chronic renal allograft interstitial fibrosis. The mouse models were established as described in the “Materials and methods” section. **A** HE, Masson trichrome staining, and Sirius Red staining stains of kidney interstitium tissue of the two groups of mice (X400, Scale bar, 100 μm). Renal parenchyma exhibiting tubular dilation (black *) and ECM deposition in interstitial space of kidney (black # and &). **B** Immunohistochemistry signals of RCAN1, YY1, E-cad, and α-SMA. Statistical analysis of the protein’s positive area was performed in different groups (*n* = 6, *p < 0.05). **C** Renal O2⋅− levels were examined by DHE staining (scale bars, 100 μm). **D** Representative blots of RCAN1.4 and EMT markers (COL1, FN, E-cad, and α-SMA) (*n* = 6). **E** Representative blots of anti-oxidant proteins (SOD1, SOD2, and Catalase) (*n* = 6). Data represent the mean ± s.e.m. *p < 0.05, as indicated.

As previously demonstrated, the expression of RCAN1.4 was remarkably inhibited in mice with chronic renal allograft IF, and overexpression of RCAN1.4 alleviated TNF-α-induced EMT in HK-2 cells by reducing oxidative stress. Therefore, we hypothesized that RCAN1.4-targeted therapy would alleviate renal allograft IF. rAAV9-RCAN1.4 was employed to infect the kidneys of mice as described in the methods. The eGFP signal in the kidneys transfected with rAAV9-RCAN1.4 indicated successful transfection (Supplementary Fig. S3A). RCAN1.4 expression was down-regulated in transplanted kidneys of the Allo group, which was reversed in the rAAV8-RCAN1.4-transfected transplanted kidneys compared to rAAV8-empty vector-treated kidneys (Fig. 3B, C). Compared with the Allo group, renal fibrosis with inflammatory cell infiltration, glomerulosclerosis, TA, and IF was improved following transfection with rAAV9-RCAN1.4 (Fig. 3A). Furthermore, overexpression of RCAN1.4 also reduced ECM deposition in the transplanted kidneys of the Allo group compared to the rAAV8-empty vector-treated kidneys (Fig. 3A). More importantly, the protein levels of FN, COL1a1, and α-SMA were also significantly down-regulated, while E-cad was upregulated, in the RCAN1.4 overexpression chronic renal allograft IF mouse model (Fig. 3D). In line with the Western blotting results, immunohistochemical staining showed a similar trend in the expression of EMT hallmarks (Fig. 3B). Consistent with the in vitro results, the chronic kidney rejection-induced reduction of anti-oxidate genes and accumulation of O2⋅− was reversed by RCAN1.4 overexpression (Fig. 3D, E). These data strongly proved that knock-in of RCAN1.4 exerted nephroprotective effects in the chronic renal allograft IF mouse model by regulating oxidative stress.

### RCAN1.4 inhibited calcineurin-NFATc1 signaling in TNF-α-induced EMT in vitro

**Fig. 4:**
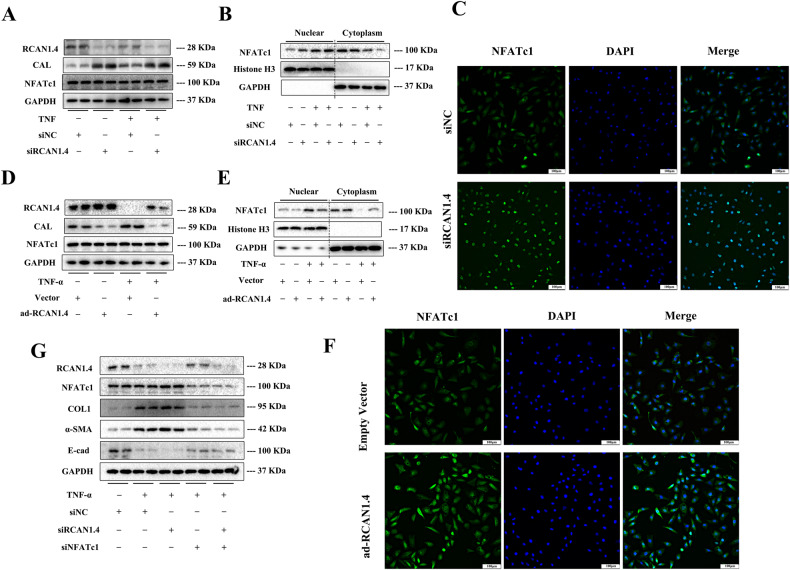
RCAN1.4 inhibited calcineurin-NFATc1 signaling in TNF-α-induced EMT in vitro. HK-2 cells were transfected with RV230-RCAN1.4 plasmid or RCAN1.4-RNAi to change the expression of RCAN1.4, followed by TNF-α stimuli. Nuclear and cytoplasmic proteins were extracted. **A**, **D** Representative blots of RCAN1.4, CAL, NFATc1, and GAPDH. **B**, **E** Representative blots of NFATc1, Histone H3, and GAPDH. **C**, **F** Fluorescence intensity was observed by fluorescence microscope (green, NFATc1; blue, DAPI). **G** Representative blots of RCAN1.4, NFATc1, and EMT markers (COL1, FN, E-cad, and α-SMA) (*n* = 3). The results are expressed as the mean ± standard error of the mean (SEM). for 3–4 independent experiments.

Since RCAN1.4 was reported to block the nuclear localization and transcriptional activity of NFAT by interacting with CaN [24, 28], we assessed CaN-NFATc1 signaling in HK-2 cells following overexpression or silencing of RCAN1.4 and TNF-α stimulation. The results showed that the knock-down of RCAN1.4 with siRNA further elevated the level of CaN in HK-2 cells treated with TNF-α, but hardly affected the expression of NFATc1 (Fig. 4A). Interestingly, in response to TNF-α stimulation, NFATc1 nuclear translocation was significantly increased in RCAN1.4-silenced HK-2 cells as compared to that in control HK-2 cells (Fig. 4B). In a parallel experiment with an immunofluorescence assay, it was also verified that NFATc1 nuclear translocation was actively regulated by siRCAN1.4 (Fig. 4C). In contrast, extensive-expression of RCAN1.4 partially inhibited nuclear translocation of NFATc1 and CaN expression, which were promoted by TNF-α stimuli (Fig. 4E, F). Similarly, the total expression of NFAT2 in cellular proteins was not influenced by the knock-in of RCAN1.4 (Fig. 4D). To further investigate the potential contribution of CaN-NFATc1 signaling in TNF-α-induced EMT, siRCAN1.4 and siNFATc1 were simultaneously employed in HK-2 cells followed by TNF-α stimulation. As shown in Fig. 4G, silencing of RCAN1.4 markedly rescued the down-regulation of E-cad and enhanced the expression of FN, COL1A1, and α-SMA, which were reversed by NFATc1 suppression. Taken together, all these studies suggest that RCAN1.4 ameliorated TNF-α-induced EMT in HK-2 cells by inhibiting CaN-NFATc1 signaling.

### YY1 modulated TNF-α induction of ROS and EMT, which was dependent on RCAN1.4

**Fig. 5:**
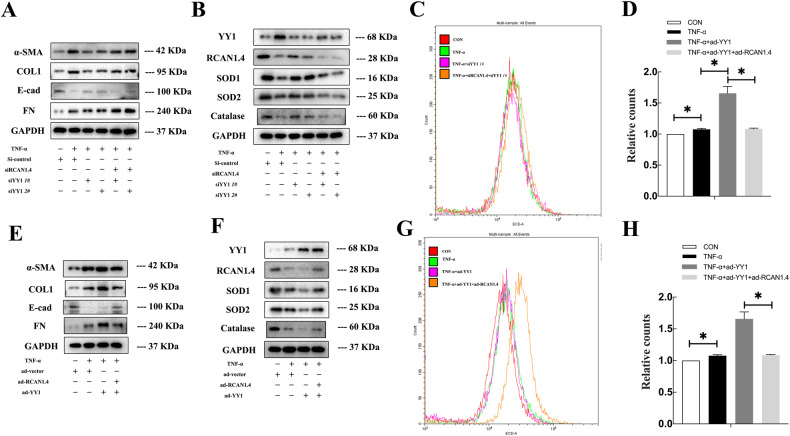
YY1 modulated TNF-α-induced reactive species and EMT dependently on RCAN1.4. **A**, **E** Representative blots of EMT markers (COL1, FN, E-cad, and α-SMA) and GAPDH (*n* = 3). **B**, **F** Representative blots of RCAN1.4, YY1, and anti-oxidant proteins (SOD1, SOD2, and Catalase). **C**, **D** (**G**, **H**) O_2_⋅− levels were detected by HE probe, and were quantified (*n* = 3), *p < 0.05, as indicated. The results are expressed as the mean ± standard error of the mean (SEM) for 3–4 independent experiments.

Since there was a noticeable boost in YY1 expression in transplanted fibrotic kidneys and TNF-α-stimulated tubular epithelial cells, we focused on the functional role of YY1 in renal allograft IF. Firstly, a reduction in E-cad and enhanced expression of FN, COL1, and α-SMA, accompanied by an apparent decline in catalase, SOD1, and SOD2, induced by TNF-α, was reversed by YY1 deficiency (Fig. 5A, B). Moreover, as shown in Fig. 5C, D, TNF-α stimulation triggered O2⋅− production, which was abolished by YY1 silencing. More importantly, the knock-down of RCAN1.4 using siRNAs partially restored the impact of siYY1 on TNF-α-induced EMT and oxidative stress in HK-2 cells (Fig. 5A–D). To further determine whether YY1 modulation of TNF-α-induced ROS and EMT is dependent on RCAN1.4, the following strategies were employed on HK-2 cells followed by TNF-α stimulation. Overexpression of YY1 further reduced E-cad expression but elevated the protein levels of FN, COL1, and α-SMA induced by TNF-α (Fig. 5E). Simultaneously, a further increased accumulation of O2⋅- and the decline in catalase, SOD1, and SOD2 induced by TNF-α stimulation were aggravated by YY1 overexpression (Fig. 5F–H). More importantly, co-overexpression of YY1 and RCAN1.4, to a certain degree, attenuated the effect of YY1 overexpression alone on TNF-α-induced EMT and oxidative stress in HK-2 cells (Fig. 5F–H). These data strongly imply that YY1 and RCAN1.4 both regulate oxidative stress and TNF-α-induced EMT in HK-2 cells in an opposing manner. Moreover, increased YY1 expression induced by TNF-α stimulation regulated ROS and EMT, which was dependent on RCAN1.4 loss.

### YY1 regulated RCAN1.4 transcription in vitro

**Fig. 6:**
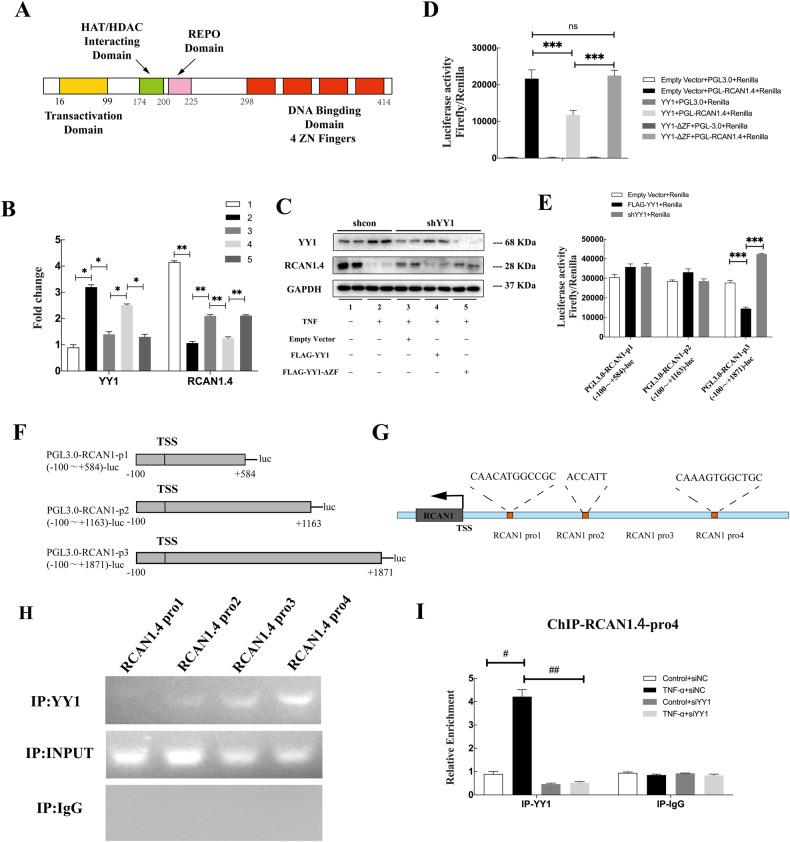
YY1 regulated RCAN1.4 transcription in vitro. **A** Schematic diagram showing YY1’s major domains. **B** mRNA levels of RCAN1.4 and YY1 were detected by qPCR assays. **C** Representative blots of RCAN1.4 and YY1. **D** and **E** HK-2 cells were co-transfected with the pGL3-RCAN1.4 promoter and YY1 overexpression plasmid, YY1-ΔZF or ShYY1, empty vector as control, along with the TK-Renilla luciferase expression plasmid. Cell extracts were assayed for luciferase activity. (*n* = 3, ***p < 0.05). **F** Schematic representation of the different lengths of the RCAN1.4 promoters, which contain various putative YY1-binding sequences, constructed to form the pGL3 luciferase vector. **G** Schematic diagram of primer pairs of the human RCAN1.4 promoter region. **H** ChIP immunoprecipitation of recruitment of YY1 to different fragments of RCAN1.4 promoter region was normalized by input. **I** The abundance of the binding levels of YY1 in the Pro4 region of the RCAN1.4 promoter was determined by ChIP in a different group (*n* = 3, ^#^*p* < 0^.^05, ^##^*p* < 0.05). The results are expressed as the mean ± standard error of the mean (SEM) for 3–4 independent experiments.

We explored the advanced mechanism by which YY1 regulates RCAN1.4 expression. As shown in Fig. 5B, the down-regulation of RCAN1.4 by TNF-α stimulation was rescued by two independent YY1 siRNAs. On the contrary, overexpression of YY1 further suppressed RCAN1.4 expression, which was inhibited by TNF-α (Fig. 5E). These results demonstrated a notable correlation between YY1 and RCAN1.4 expression. Since YY1 was reported as a ubiquitous and crucial transcription factor that may regulate gene expression in a variety of cellular settings [29], we supposed that YY1 reduced RCAN1.4 expression through transcriptional regulation. To test our hypothesis, we transfected YY1 knockdown HK-2 cells with YY1 wild-type (YY1 WT) or ZN finger domain-deleted (YY1-ΔZF) expression plasmids according to the structure of YY1, followed by TNF-α stimulation (Fig. 6A). As shown in Fig. 6B, C, YY1 overexpression suppressed RCAN1.4 expression at the mRNA and protein level. More importantly, YY1-ΔZF did not repress RCAN1.4 expression in YY1 knockdown cells as it did in YY1 WT cells subjected to TNF-α stimulation (Fig. 6B, C), indicating that YY1-mediated inhibition of RCAN1.4 is reliant on its DNA binding domain activity.

To verify the mechanisms by which YY1 mediates RCAN1.4 transcriptional regulation in HK-2 cells with TNF-α-induced EMT, a dual-luciferase reporter assay using a pGL3-RCAN1.4-luc reporter system was performed. Firstly, we cloned the RCAN1.4 whole promoter fragment, which was then engineered into the pGL3 basic luciferase reporter vector. After being transfected into HK-2 cells with various plasmids, including YY1-ΔZF, YY1 WT, or empty vector, luciferase activity was detected. As indicated in Fig. 6D, RCAN1.4 transcription was inhibited by YY1 overexpression and restored by YY1-ΔZF in HK-2 cells, which was in line with the RT-PCR and WB results. Next, we identified different candidate binding sites of the RCAN1.4 core promoter region corresponding to YY1 binding from the JASPAR database (Supplementary Fig. S4A). To this end, we cloned three pGL3 basic luciferase reporter vectors of different fragments with RCAN1.4 promoter lengths. RCAN1.4 transcription was suppressed by YY1 WT and rescued by YY1 depletion in HK-2 cells in the pGL3-RCAN1.4-P3-luc system (Fig. 6E). By comparison, neither RCAN1.4-P1 nor RCAN1.4-P2 luciferase activity was detected (Fig. 6E). Furthermore, using a mutant sequence of RCAN1.4-P3, we created a mutant luciferase reporter to substantiate the interaction between the P3 fragment and YY1 (Supplementary Fig. S4B). The results showed no interaction between the mutant RCAN1.4-P3 fragment and YY1 (Supplementary Fig. S4C).

To further confirm the YY1-induced transcriptional regulation of RCAN1.4, a parallel ChIP experiment was performed. According to the three YY1-binding regions of the RCAN1.4 promoter predicted by the JASPAR database, we constructed a series of primers. Three predicted regions (pro1, 2, 4) and a negative control region (pro3) 2000 bp upstream of the transcriptional initiation site of RCAN1.4 were detected, and endpoint-PCR assays were performed. As shown in Fig. 6F, ChIP performed with the YY1 antibody was greatly enriched for the RCAN1.4 promoter 4 region. In parallel, we observed elevated YY1 recruitment in the RCAN1.4 promoter 4 region in HK-2 cells treated with TNF-α, whereas the YY1 silencing showed the opposite result (Fig. 6G). These data strongly proved that YY1 inhibits the RCAN1.4 transcription by interacting with the RCAN1.4 promoter 4 region.

### Cooperation of YY1 with HDACs suppressed RCAN1 transcription

**Fig. 7:**
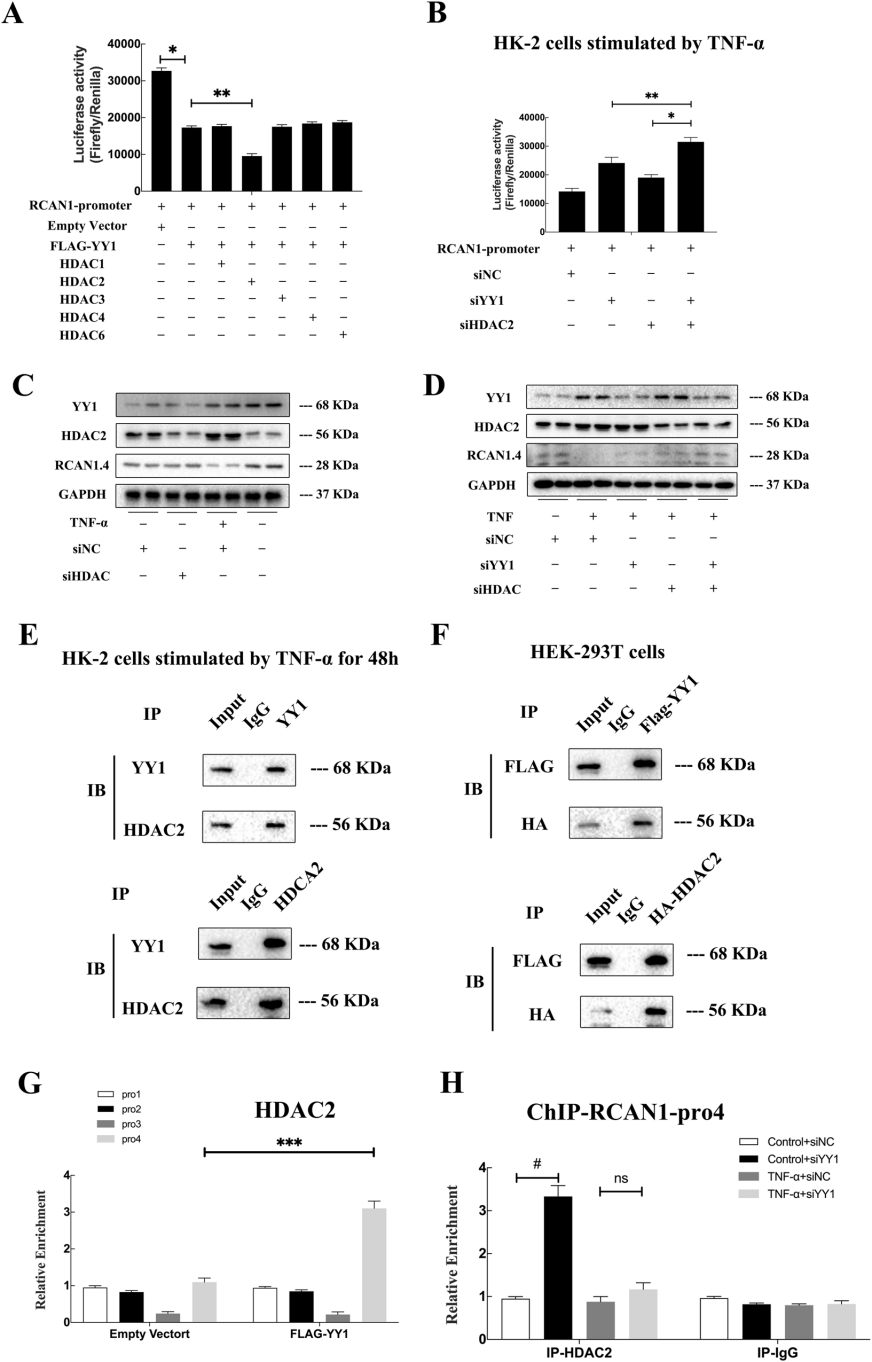
Cooperation of YY1 with HDACs suppressed RCAN1 transcription. **A** pGL3-RCAN1.4 promoter, FLAG-YY1, various HDACs plasmids, and empty vector as control, along with the TK-Renilla luciferase expression plasmid were co-transfected into HK-2 cells. 24 h after transfection, cell extracts were assayed for luciferase activity (*n* = 3, *p < 0.05, **p < 0.05). **B** pGL3-RCAN1.4 promoter, siYY1, and siHDAC2 along with the TK-Renilla luciferase expression plasmid were co-transfected into HK-2 cells, followed by TNF-α stimuli for 24 h, cell extracts were assayed for luciferase activity (*n* = 3, *p < 0.05, **p < 0.05). **C**, **D** Representative blots of YY1, HDAC2, RCAN1.4, and GAPDH. **E** co-IP analysis of YY1 and HDAC2 in immunoprecipitates of HK-2 cells, followed by TNF-α stimuli for 48 h. **F** co-IP analysis of YY1 and HDAC2 in immunoprecipitates of HEK293T cells co-transfected with Flag-tagged YY1 plasmids and HA-tagged HDAC2 plasmids. **G** The abundance of the binding levels of YY1 in the 4 regions of the RCAN1.4 promoter was determined by ChIP (*n* = 3, ***p < 0.05). **H** The abundance of the binding levels of HDAC2 in the Pro4 region of the RCAN1.4 promoter was determined by ChIP in different groups (*n* = 3, #*p* < 0.05). The results are expressed as the mean ± standard error of the mean (SEM) for 3–4 independent experiments.

The main domain of the YY1 protein interacts with histone deacetylases (HDACs), which serve as epigenetic co-repressors to inhibit gene expression [30]. Thus, we determined whether HDACs had synergism with YY1 in RCAN1.4 transcriptional repression using a dual-luciferase reporter assay and Western blotting. Firstly, we constructed various overexpression plasmids of HDAC isoforms to detect their effect on RCAN1.4 promoter activity. The luciferase assay results confirmed that only HDAC2 significantly aggravated the effect of YY1-mediated RCAN1.4 repression, whereas no obvious distinct effects on RCAN1.4 promoter activity were shown in the other four HDACs (Fig. 7A). Next, to further understand the crucial role of HDAC2 in HK-2 cells stimulated by TNF-α, the following experiments were carried out. As indicated in Fig. 7C, D, RCAN1.4 expression was reduced by TNF-α, which was then restored by HDAC2 silencing and YY1 silencing. Furthermore, co-transfection with the HDAC1 siRNA and YY1 siRNA further restored RCAN1.4 expression, as assessed by Western blotting (Fig. 7C, D). Of note, HDAC2 silencing had little impact on the expression of YY1 (Fig. 7C). As with the Western blotting, the luciferase reporter assay showed the same results (Fig. 7B). In addition, co-IP experiments were performed on HK-2 cells treated with TNF-α for 48 h. The results suggested that YY1 directly interacted with HDAC2 (Fig. 7E). A consistent result was obtained in HEK293T cells transfected with the overexpressing plasmid HA-HDAC2 and Flag-YY1 (Fig. 7F). In order to reconfirm HDAC2 co-repressors on RCAN1.4 promoter, further ChIP assays were carried out. The results suggested that YY1 and HDAC2 were strongly recruited to the RCAN1.4-pro4 region together (Fig. 7G). Moreover, YY1 silencing also decreased the recruiting of HDAC1 to the RCAN1.4-pro4 region (Fig. 7H). Taken together, these findings strongly confirm that YY1 and HDAC2 form a multi-molecular complex and are involved in TNF-α-induced RCAN1.4 transcriptional repression.

## Discussion

In the current research, we demonstrated a significant and novel mechanism by which RCAN1.4 regulated chronic allograft dysfunction (CAD) in mice. In addition, YY1 and HDAC2 synergistically mediated RCAN1.4 expression was essential in modulating ROS production, such as O2⋅−, and IF/TA formation in transplanted kidneys and in TNF-α-induced EMT in vitro. We also demonstrated that RCAN1.4 over-expression may alleviate IF/TA with CAD by blocking the CaN/NFATc1 signaling pathway and decreasing the ROS level.

As the fundamental cells of the renal tubulointerstitium, renal tubular epithelial cells have been implicated as important participants in allograft failure and kidney injury development [4]. When tubular epithelial cells were stimulated with several inflammatory cytokines, including TNF‐α, interleukin (IL)-1β, and IL-6, EMT was facilitated in the development of allograft kidney IF; however, the underlying mechanisms remained ambiguous [11, 14, 31]. Our previous study verified that TNF-α could induce EMT in HK-2 cells through activating Akt/Smurf2 signaling and participated in the development of IF/TA in renal allografts of CAD patients [14, 32]. Simultaneously, Zhao reported that TNF-α promoted the generation of ROS, such as O2⋅− and ⋅OH. More importantly, Wang et al. reported that oxidative stress was one of the key factors in CAD IF/TA, which leads to EMT in vivo and stimulates rat renal tubular epithelial cells to undergo induction of EMT in vitro [20]. The findings of these studies were in line with ours, demonstrating that TNF-α induced EMT in HK-2 cells by promoting oxidative stress.

As a specific endogenous inhibitor of CaN, RCAN1 participated in multifarious biological functions, including regulating vascular endothelial growth factor (VEGF)-mediated tubular morphogenesis in endothelial cells, preventing proliferation and migration of cancer cells and defending against calcium-mediated oxidative stress [33, 34]. In addition, RCAN1.4 was reported to be associated with various fibrotic diseases. For instance, Pan et al. indicated that excessive expression of RCAN1.4 could reduce ECM protein accumulation by inhibiting the CaN/NFAT3 signaling pathway in carbon tetrachloride (CCI_4_)-induced liver fibrogenesis [35]. Another report by Chen et al. demonstrated that mitochondrial fragmentation was increased in mesangial cells as a result of high glucose-induced RCAN1.4 upregulation [36]. Our previous study proved that RCAN1.4 attenuates UUO-mediated renal fibrosis by inhibiting CaN-mediated nuclear translocation of NFAT2 [24]. However, the role of RCAN1.4 in CAD IF/TA remained to be determined. Herein, we found that RCAN1.4 expression was down-regulated in transplanted kidneys with IF/TA progression, whereas overexpression of RCAN1.4 attenuated CAD IF/TA and reversed the expression of EMT markers, including COL1, α-SMA, FN, and E-Cad in mouse chronic renal allograft IF models in vivo and in TNF-α-induced EMT in HK-2 cells in vitro. In addition, many studies have confirmed that RCAN1 inhibits tumor growth and migration by targeting the CaN/NFAT pathway [33, 37]. Besides, Peiris et al. reported that RCAN1 altered mitochondrial function and increased the neurons’ susceptibility to oxidative stress in Down’s syndrome and Alzheimer’s disease, which indicated that the expression of RCAN1 was related to oxidative stress levels [23]. Similar to the role of RCAN1.4 in various disease models, we found that RCAN1.4 alleviated the TNF-α-induced EMT in vitro via the CaN/NFAT1c pathway, and subsequently blocked the nuclear translocation of NFAT1c. Interestingly, increasing RCAN1.4 expression did not aggravate TNF-α stimulated-ROS generation, but activated multiple endogenous antioxidants, including SOD1, SOD2, and catalase both in vivo and in vitro, resulting in the alleviation of oxidative stress. These results may be explained by Chen’s study, which demonstrated that RCAN1.4 could stimulate mitochondrial fission, subsequently promoting the accumulation of ROS in diabetic kidney disease [36].

The specific mechanism of RCAN1.4 in various diseases is complicated. Recently, the study of epigenetic modifications of the RCAN1.4 gene has become increasingly fervent. In breast cancer, Deng et al. elucidated that RCAN1.4 was transcriptionally driven in a super-enhancer manner by the transcription of RUNX family transcription factor 3 (RUNX3), which acted as an oncogene to promote tumor growth [38]. Simultaneously, Pan et al. illustrated that DNMT1 and DNMT3b methyltransferases bind to RCAN1.4 promoter and inhibit RCAN1.4 expression in liver fibrogenesis [35]. Moreover, another report by Li et al. emphasized the importance of DNA methylation patterns of RCAN1.4 modified by HIV-1 infection in diabetic kidney disease [25]. However, the novel molecular mechanism for RCAN1.4 regulation in allograft kidney IF remains to be determined. Since various transcription factors are activated in renal disease, followed by inhibition or activation of many target genes, we predicted several potential transcription factors that may bind to the RCAN1.4 core promoter region. Our study showed that RCAN1.4 was transcriptionally regulated by YY1. Emerging evidence indicates that YY1, a multifunctional zinc finger DNA binding transcription factor, is involved in both cancer and kidney disease, including CKD and diabetic nephropathy [26, 27]. Previously, the role of YY1 in the development of renal lesions in different kidney diseases has been illustrated, although the mechanism differed. Yang et al. reported that YY1 was up-regulated and induced EMT in diabetic nephropathy-induced renal fibrosis [27]. Du et al. demonstrated that the enhanced level of YY1 acetylation was regulated by Sirt1 reduction in diabetic nephropathy [39]. Our study confirmed that YY1 regulated TNF-α-induced EMT in HK-2 cells through the modulation of ROS generation. Furthermore, in-depth ChIP assays and luciferase reporter assays were performed to prove that YY1 was a transcription factor candidate for binding to the core promoter region of RCAN1.4. As a ubiquitously expressed transcription factor, YY1 usually does not work alone; it recruited various transcription regulators and epigenetic modification regulators, which determined its gene transcriptional silencing or stimulating mechanisms. Since the HDAC interacting domain is one of the key domains of YY1, we assessed the role of HDACs in YY1-mediated inhibition of RCAN1.4 transcription. Our in vitro study confirmed that HDAC2, instead of other HDACs, could cooperate with YY1 and significantly catalyzed the effect on YY1-mediated RCAN1.4 repression. Furthermore, the specific promoter region of RCAN1.4 interacted with HDAC2, which was dependent on YY1 binding sites on the RCAN1.4 promoter. We proved that YY1 acts as an inhibitor of RCAN1.4 expression with HDAC2 interaction, formed a multi-molecular complex, and was recruited to the RCAN1.4 promoter during TNF-α-induced EMT in HK-2 cells. Identifying these inhibitory molecules responsible for YY1-mediated RCAN1.4 inhibition is crucial for deeply understanding the epigenetic regulation during transplanted renal IF/TA progression in CAD patients.

## Conclusions

In summary, we demonstrated a significant and novel mechanism that linked YY1 to RCAN1.4 in IF/TA formation in CAD patients. RCAN1.4, modulated by the multi-molecular complex of YY1/HDAC2 in an epigenetic manner, mediated a nephroprotective effect partly through modulating O2⋅− generation and the CaN/NFATc1 pathway. Therefore, the YY1-RCAN1.4 axis may be a novel therapeutic target for the treatment of IF/TA in CAD patients.

## Materials and methods

### Animal preparation

Male adult C57BL/6J mice and BALB/c mice (22–30 g) were provided by the Laboratory Animal Center of Nanjing Medical University (Nanjing, China). All the mice had access to eat and drink ad libitum in a pathogen-free environment at the appropriate temperature and humidity. All experiments were authorized by the Ethics Committee of Nanjing Medical University (Nanjing, China), and the procedures were carried out in adherence to the Animal Research Ethics Committee of the Nanjing Medical University (Nanjing, China).

### Mouse model of chronic renal allograft IF

After inhalation anesthesia with isoflurane, a mid-abdominal incision was made on C57BL/6 mice, and the left ureter was carefully dissociated with microvascular forceps. The left kidney and left renal artery and vein were dissociated and perfused in situ with 4 °C heparin saline (80 U/ml) from the abdominal aorta at the level of the lower end of the renal artery until the kidney became khaki-yellow. The kidney artery was then severed, followed by cutting off the ureter at the entrance of the bladder, and storage of the donor kidney in Belzer UW^®^ cold storage solution at 4 °C. Resection of the left kidney was performed on the recipient BALB/c mice after anesthesia. The vascular cavity was then rinsed with heparin water, and the repaired donor kidney was placed beside the freed vascular segment. End-to-side anastomosis of the renal artery and abdominal aorta was performed with an 11–0 suture. In addition, the ureteral stump was embedded in the recipient’s bladder. Antibiotics (penicillin) were intraperitoneally injected once a day for the first 3 days after the operation. A liquid diet was given on the second day after the operation, and Tacrolimus (1 mg/kg) was given intraperitoneally every day for the first week and every week for the second to fourth week after the operation. This procedure was performed to establish a mouse model of chronic renal allograft IF (Allo group). Simultaneously, the left kidney of wild-type BALB/c mice were transplanted into wild-type BALB/c mice to establish a sham operation group (Syn group). All the mice were sacrificed several weeks after the operation, and the transplanted kidney tissues were collected and routinely stained.

### Recombinant adeno-associated virus (rAAV9)-mediated RCAN1.4 over-expression in mice

The rAAV9-packed RCAN1.4 over-expression plasmid labeled with the green fluorescent protein (GFP), provided by ViGene Biosciences, Inc (Jinan, China), was used to upregulate RCAN1.4 expression in mouse kidneys. Donor mice were transfected with rAAV9-packed RCAN1.4 (100 μl, 1 × 10^12^ [v g/mL]) via the caudal vein. Two weeks later, kidney transplantation operations were performed on mice, as described above. In addition, some recipient mice were treated with rAAV9-packed RCAN1.4 again at 2 weeks and 6 weeks after the operation.

### Cell culture

The human renal tubular epithelial cells (HK-2) and HEK293T cells were provided by the American Type Culture Collection (ATCC, Manassas, VA). Briefly, the cells were cultured in modified Eagle’s medium (MEM) (Invitrogen, USA) supplemented with 10% fetal bovine serum (FBS, Gibco, USA) and 1% penicillin-streptomycin at 37 °C with 5% CO_2_. TNF-α (0–100 ng/ml, Peprotech, USA) was administered to HK-2 cells for 48 h to induce EMT. In addition, cells in some specific groups were pretreated with acetylcysteine (NAC, 1 mmol/L, MedChemExpress, USA) and/or H_2_O_2_ (100 μmol/L) before TNF-α stimulation.

### Transfection in vitro

For RNA interference, various siRNAs were employed on HK-2 cells to silence target genes, including RCAN1.4, YY1, and NFATc1, for 36 h with using Lipofectamine 3000 reagent (Invitrogen, USA). All siRNAs were synthesized by KeyGen BioTECH (Nanjing, Jiangsu, China) and the sequences used are listed in Table 1. For RCAN1.4 and YY1 over-expression in vitro, HK-2 cells were transfected with a lentivirus-packed and GFP-labeled over-expression RCAN1.4 or YY1 plasmid (ViGene Biosciences, Inc, Jinan, China) at a concentration of 1 × 10^7^ TU/ml for 24 h, followed by puromycin selection (6 μg/ml, Beyotime, Shanghai, China). In addition, for stable knockdown of YY1, a short hairpin RNA (shRNA) targeting YY1 (shYY1) (provided by Sigma-Aldrich, USA), was employed to transfect HK-2 cells.

**Table 1:** siRNA sequences.

Genes	Sequences
RCAN1.4	5′-CUGUGUGGCAAACAGUGAU-3′
YY1 si#1	5′-CCUCCUGAUUAUUCAGAAUTT-3′
YY1 si#3	5′-GCUCCAAGAACAAUAGCUUTT-3′
NFATc1	5′-GAGUCUCUCAGUUCAGUGU-3′

### Histopathology and immunohistochemistry

Kidney samples were placed in 4% paraformaldehyde, followed by being segmented at a thickness of 4 μm from paraffin-embedded tissues. Then hematoxylin and eosin (H&E) were carried out to assess pathological renal injury, and Masson’s trichrome staining and Sirius Red staining to observe collagen deposition. Renal tubule injury severity was determined with five grades (0–4): 0, no obviously visible injury; 1, injury <25%; 2, injury 25–50%; 3, injury 50–75%; and 4, injury >75% [40–42].

As for immunohistochemical staining, antibodies, included anti-RCAN1 (1:200, D6694, Sigma-Aldrich), anti-YY1 (1:200, ab109228, Abcam), anti-E-cad (1:100, #40860, SAB), anti-TNF-α (1:200, ab6671, Abcam), anti-α-SMA (1:100, 14395-1-AP, Proteintech), were employed to incubate kidney section, followed by an appropriate secondary antibody. The stained samples were observed using an Ortho microscope (OLYMPUS, Tokyo, Japan).

### Renal function measurement

In order to assess mouse renal function, commercial assays were used to detect blood urea nitrogen (BUN) and creatinine (Cr) (Changchunhuili Biotech Co, Changchun, China).

### Western blot analysis

Briefly, protein samples, extracted from mouse kidneys and HK-2 cells, were separated on SDS–PAGE gels and PVDF membrane. PVDF membranes were then blocked for 2 h with 5% non-fat milk before being incubated at 4 °C overnight with primary antibodies included RCAN1 (1:1000, D6694, Sigma-Aldrich), YY1(1:100, sc-7341, SANTA CRUZ), E-cad (1:1000, #40860, SAB), COL1A1 (1:1000, A1352, Abclonal), αSMA (1:5000, #40482, SAB), FN (1:1000, 610077, BD Bioscience), HDAC2 (1:1000, 12922-3-AP, Proteintech), SOD1 (1:1000, 10269-1-AP, Proteintech), SOD2 (1:2000, 24127-1-AP, Proteintech), Catalase (1:2000, 21260-1-AP, Proteintech), Histone H3 (1:1000, 9715s, CST), and GAPDH (1:1000, 60004-1-Ig, Proteintech). On the second day, the PVDF membranes were incubated with an appropriate secondary antibody for 2 h at 37 °C after being washed three times with tris buffered saline-tween (TBST). Goat anti-mouse and goat anti-rabbit secondary antibodies were obtained from Proteintech (1:5000, SA00001-1, SA00001-2). Using ImageJ software (NIH, Bethesda, MD, USA), band densities were calculated. In addition, nuclear and cytoplasmic protein extraction Reagents (78833) were obtained from Thermo Fisher Scientific.

### Immunofluorescence

Cells were seeded on cell climbing sheets, fixed with 4% paraformaldehyde. After blocking with QuickBlock™ Blocking Buffer (P0260, Beyotime), cells were given primary antibodies to incubate, including RCAN1 (1:200, D6694, Sigma-Aldrich), YY1 (1:200, ab109228, Abcam), and NFATc1 (1:100, 66963-1-Ig, Proteintech). On the second day, Alexa Fluor 488 goat anti-mouse IgG (H + L) or Alexa Fluor 594 goat anti-rabbit IgG (H + L) secondary antibodies were employed to incubate cells, followed by nuclear staining (0100-20, SouthernBiotech) for 15 min. The fluorescence signals were analyzed by the thunder imager fast high-resolution inverted fluorescence imaging system (THUNDER DMi8, Leica, German).

### Measurement of ROS

Hydroethidine (HE, 10 μM, 20 min), obtained from MedChemexpress (HY-D0079, New Jersey, America), was employed to detect O2⋅- production [43]. HE was observed by flow cytometer at Ex/Em 518/616 nm. As for ROS in vivo, dihydroethidium (DHE, 5 μmol/l, Sigma, USA) was employed to stain renal frozen sections, subsequently incubated at 37 °C for 30 min.

### Quantitative real-time polymerase chain reaction (qRT-PCR)

With TRIzol Reagent (#15596018, Invitrogen), total cellular RNA was extracted, followed by reverse transcription into cDNA using the HiScript II Q RT SuperMix Kit (R223-01, Vazyme, Nanjing, China). Real-time fluorescent quantitative PCR was performed by StepOne™ Real-Time PCR System using SYBR-Green master mix (#RR820B, Takara). The 2^−ΔΔCt^ method was used to measure relative mRNA levels after being normalized to GAPDH. The primer sequences are listed in Table 2.

**Table 2:** qPCR primer sequences.

Genes	Sequences
RCAN1.4	Forward: 5′-TTTAGCTCCCTGATTGCCTGT-3′
	Reverse: 3′-AAAGGTGATGTCCTTGTCATACG-5′
YY1	Forward: 5′-ACGGCTTCGAGGATCAGATTC-3′
	Reverse: 3′-TGACCAGCGTTTGTTCAATGT-5′
GAPDH	Forward: 5′-GTCAAGGCTGAGAACGGGAA-3′
	Reverse: 5′-AAATGAGCCCCAGCCTTCTC-3′

### Immunoprecipitation (IP) and chromatin immunoprecipitation (ChIP) assay

For co-IP, a specific assay (abs955, Absin, Shanghai, China) was used. In brief, total cell lysates were mixed with 20 μl protein A/G-agarose beads at 4 °C for 1 h. Then, 5 μl anti-YY1 was added into the supernatant and incubated overnight. After that, 10 μl protein A/G-agarose beads were employed for 3 h. After centrifuging, the beads were then boiled with an SDS-loading buffer. The immunoprecipitated samples were collected for immunoblot detection with anti-YY1 antibody or anti-HDAC2 antibody.

ChIP assays were performed in accordance with the protocol (53008, Active Motif, USA). Briefly, cells were pretreated to fix protein/DNA interactions, followed by shearing of the fixed chromatin by sonication (10 pulses of 20 s each, with a 30 s rest). The sheared chromatin was then incubated with an anti-YY1, anti-HDAC2, or control IgG (#2729S, CST). The captured chromatin was then eluted, the cross-links were reversed, and the recovered DNA was purified using a Chromatin IP DNA Purification kit (58002, Active Motif, USA). In addition, qRT-PCR was performed on the purified DNA to identify the association with the protein. The specific primers are listed in Table 3.

**Table 3:** ChIP-qPCR primer sequences.

Primer	Position and length(bp)	Sequences
RCAN1-pro1	188–353----------166	F 5′-TTTAGCTCCCTGATTGCCTGT-3′
		R 3′-CCCTCAACCTTGGCAAAATA-3′
RCAN1-pro2	1030–1189-------160	F 5′-CTCCAAAGAACCCAGTGCAT-3′
		R 3′-ACAGTGAGAACTTGGGTCTGAA-3′
RCAN1-pro3	1497–1657--------161	F 5′-TGTCTCGCTTAAATTCCCAAA-3′
		R 3′-AGTTCCGCTTTTCTGACAGC-3′
RCAN1-pro4	1584–1738--------155	F 5′-AGCTGTCAGAAAAGCGGAAC-3′
		R 3′-GTTATTGTACCCCGCCCTTC-5′

### Luciferase reporter assays

According to the different candidate binding sites of the RCAN1.4 core promoter region corresponding to YY1 binding from the JASPAR database, the RCAN1.4 promoter reporter (pGL3-RCAN1.4-Luc) was constructed by Tsingke Biotechnology. For the transcriptional activity assay, HK-2 cells or 293T cells were seeded into 12-well plates and co-transfected with the pGL3-RCAN1.4 promoter (−100 to +584/+1163/+1871) reporter plasmids, TK-Renilla luciferase plasmid, and the YY1 plasmid or control plasmid using Lipofectamine 3000 (Invitrogen). Two days later, the luciferase activity was measured with a Dual-Luciferase Reporter Assay System (# E1910, Promega). Renilla luciferase was employed to normalize the firefly luciferase activity.

### Statistical analysis

All data are expressed as mean ± standard error of the mean (SEM). Statistical analyses involved one-way analysis of variance (ANOVA) and the Student–Newman–Keuls test. *p*-value < 0.05 was accepted as statistically significant.

## Supplementary information

Supplementary figure legends

Supplementary fig 1

Supplementary fig 2

Supplementary fig 3

Supplementary fig 4

Original Data File

## Data Availability

The datasets used and/or analyzed during the current study are available from the corresponding author upon reasonable request.
