# Supplementary material for: Disruption of RCAN1.4 expression mediated by YY1/HDAC2 modulates chronic renal allograft interstitial fibrosis
Source: Cell Death Discov. 2023 Jul 28;9:271. doi: 10.1038/s41420-023-01574-z (PMC10382480; doi:10.1038/s41420-023-01574-z)
Supplement: Supplementary file 1 — Supplementary figure legends [file 41420_2023_1574_MOESM1_ESM.docx]

**Supplementary Figure 1. RCAN1.4 expression was down-regulated in vivo and in vitro**. The mouse renal transplanted models of chronic renal allograft interstitial fibrosis were established as described in methods and materials. (A-B) BUN and Cr levels were evaluated in mice exposed to kidney transplantation surgery (n=6, *P<0.05). (C) Western blotting analysis was performed after collecting kidney lysates. Representative blots of EMT markers (COL1, FN, E-cad and α-SMA) (n=6). (D) Representative blots of anti-oxidant proteins (SOD1, SOD2 and Catalase) (n=6). (E) Renal O2⋅- levels were examined by DHE staining (Scale bars, 100μm). In vitro, TNF-α(0-100ng/mL) was applied to HK‐2 cells to induce EMT. (F) mRNA levels of RCAN1.4, YY1 were detected by qPCR (n=3). (G-H) O2⋅- levels were detected by HE probe, and were quantified(n=3). (I) Representative blots of anti-oxidant proteins in vitro(SOD1, SOD2 and Catalase) (n=3). The results are expressed as the mean ± standard error of the mean (S.E.M.). for 3–4 independent experiments. ^*^p<0.05, ^**^p<0.05as indicated.

**Supplementary Figure 2.** The level of reactive species induced by TNF-α stimuli in vitro. (A-B) O_2_⋅- levels were detected by HE probe, and were quantified(n=3). (C) Representative blots of anti-oxidant proteins in vitro (SOD1, SOD2 and Catalase) (n=3). The results are expressed as the mean ± standard error of the mean (S.E.M.). for 3–4 independent experiments. ^*^p<0.05, as indicated.

**Supplementary Figure 3. Recombinant adeno-associated virus-mediated overexpression of RCAN1.4 in mouse kidney**. Donor mice were slowly injected with rAAV9-packed RCAN1.4 (100ul, 1×10^12^ v.g/mL/mouse) through tail mainline. Two weeks later, kidney transplantation operations were performed on mice as described above. In addition, some recipient mice were treated with rAAV9-packed RCAN1.4 again at two weeks and six weeks after operation (A) Frozen section of kidneys were taken at 16 weeks after kidney transplantation. Scale bars, 100μm, n=6

**Supplementary Figure 4. YY1 regulated RCAN1.4 transcription in vitro**. (A)YY1-binding regions of RCAN1.4 promoters predicted by JASPAR database. (B) Schematic representation of pro4 region of the RCAN1.4 containing the wild-type motif binding sequence or the mutant type. (C)pGL3-RCAN1.4-p3, PGL3-RCAN1.4-MUT-p3, FLAG-YY1, empty vector as control, along with the TK-Renilla luciferase expression plasmid were co-transfected into HK-2 cells. 24 hours after transfection, cell extracts were assayed for luciferase activity (n=3, ^###^p<0.05).
