# Supplementary figures and images for: Disruption of RCAN1.4 expression mediated by YY1/HDAC2 modulates chronic renal allograft interstitial fibrosis
Source: Cell Death Discov. 2023 Jul 28;9:271. doi: 10.1038/s41420-023-01574-z (PMC10382480; doi:10.1038/s41420-023-01574-z)

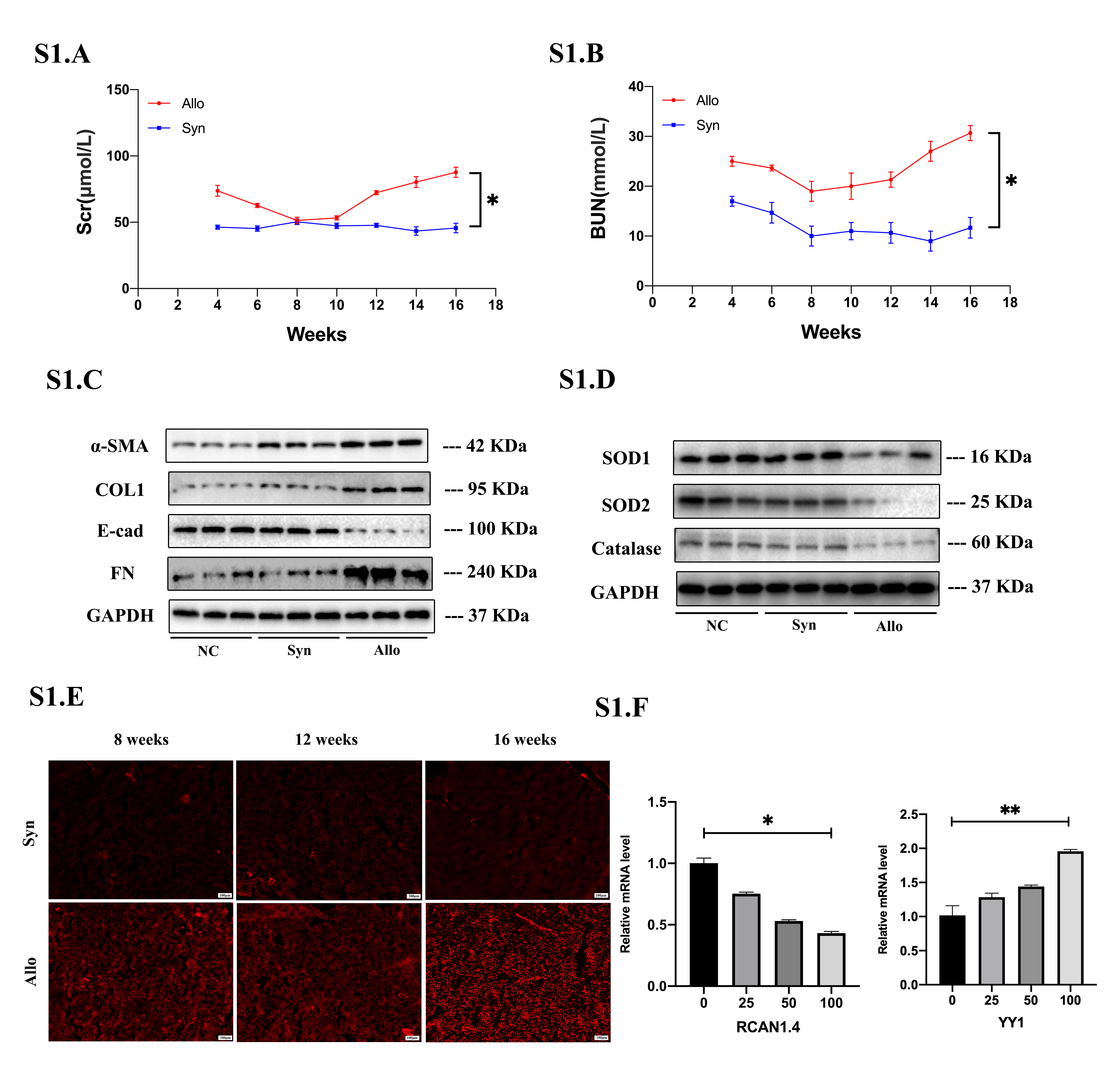

Supplement: Supplementary file 2 — Supplementary fig 1 [file 41420_2023_1574_MOESM2_ESM.tif]

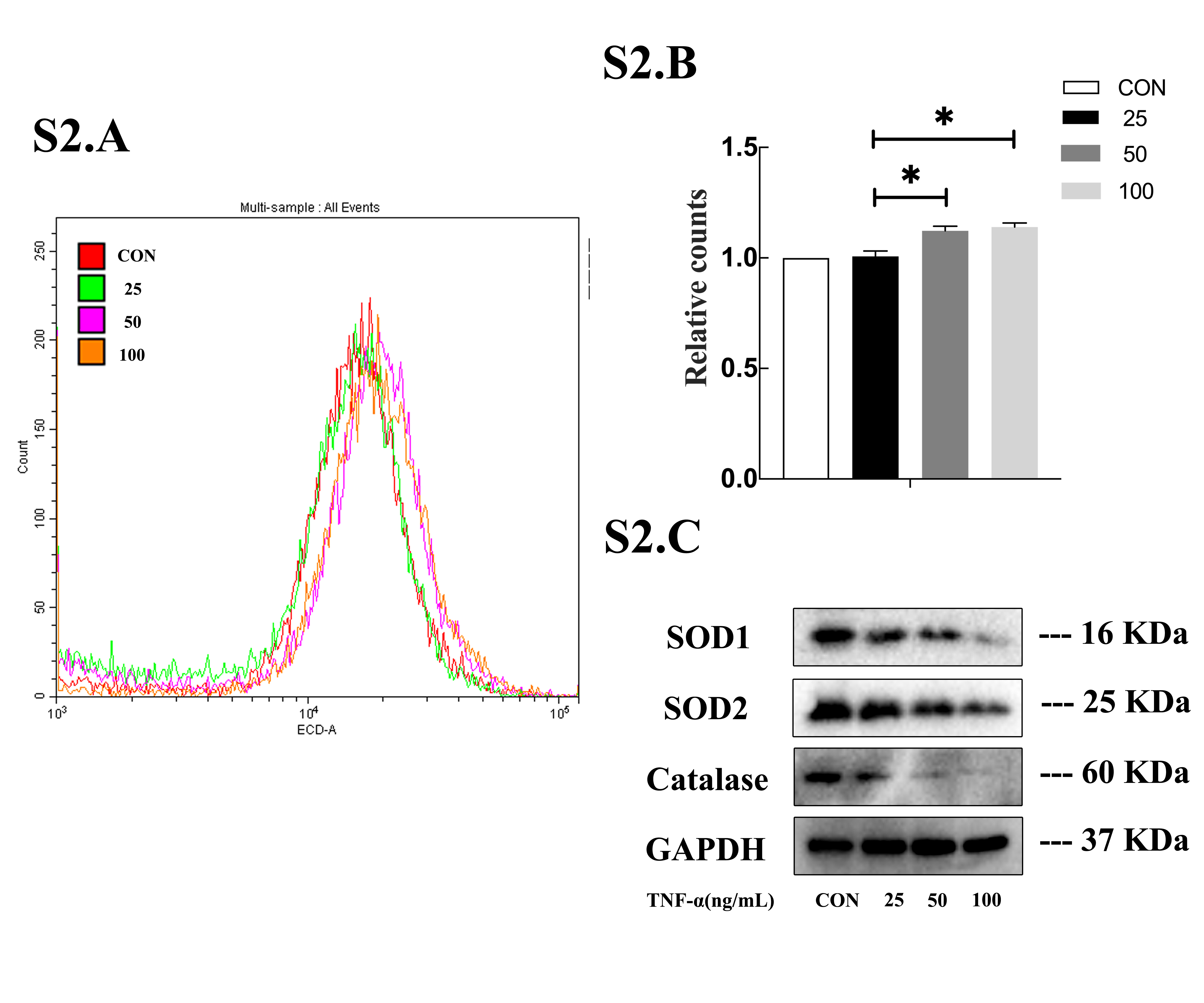

Supplement: Supplementary file 3 — Supplementary fig 2 [file 41420_2023_1574_MOESM3_ESM.tif]

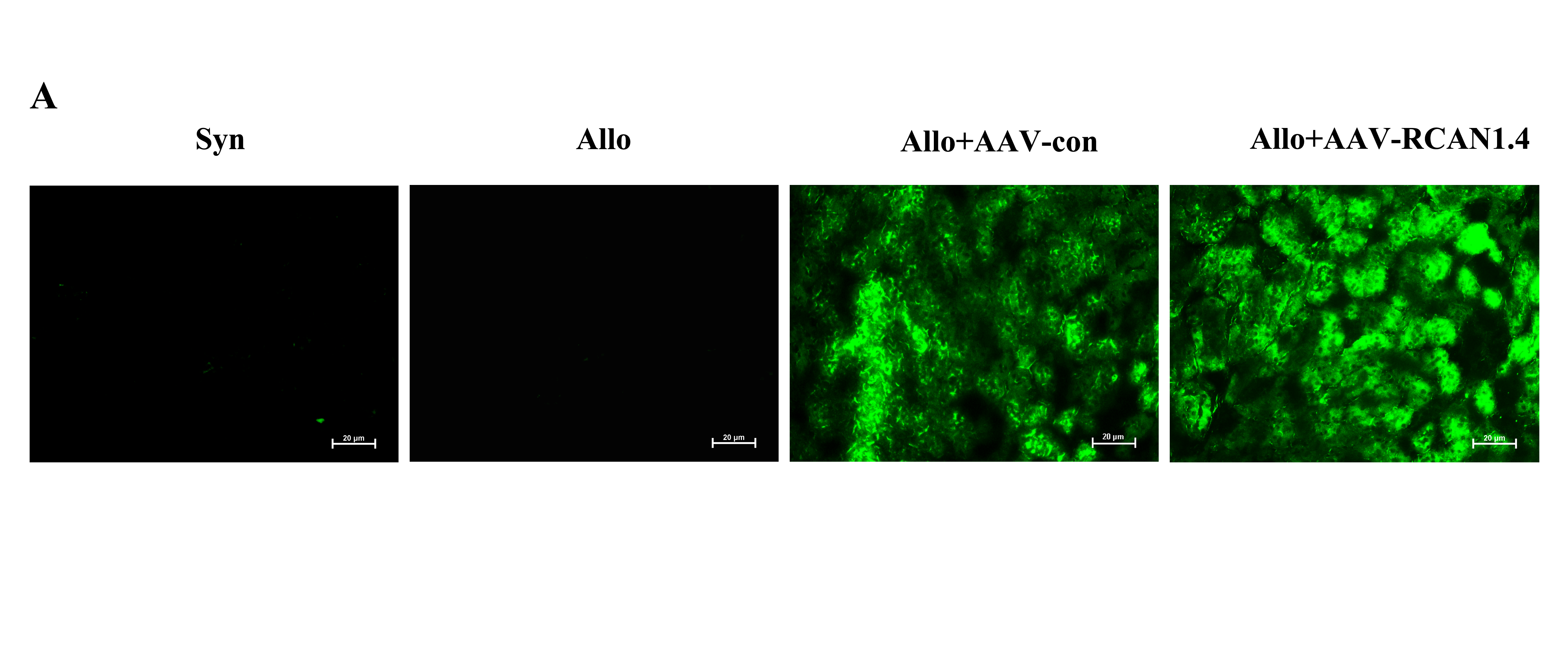

Supplement: Supplementary file 4 — Supplementary fig 3 [file 41420_2023_1574_MOESM4_ESM.tif]

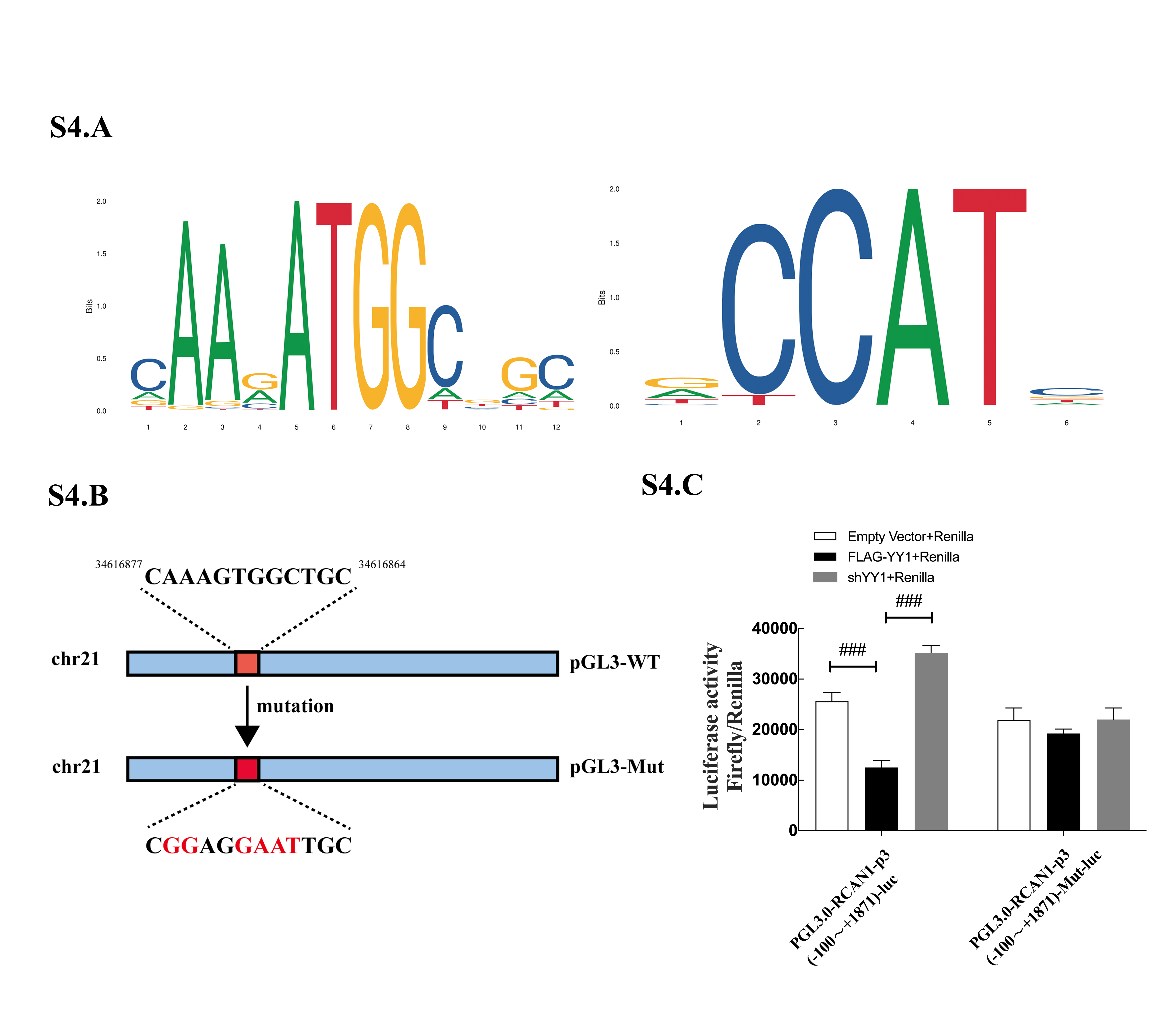

Supplement: Supplementary file 5 — Supplementary fig 4 [file 41420_2023_1574_MOESM5_ESM.png]

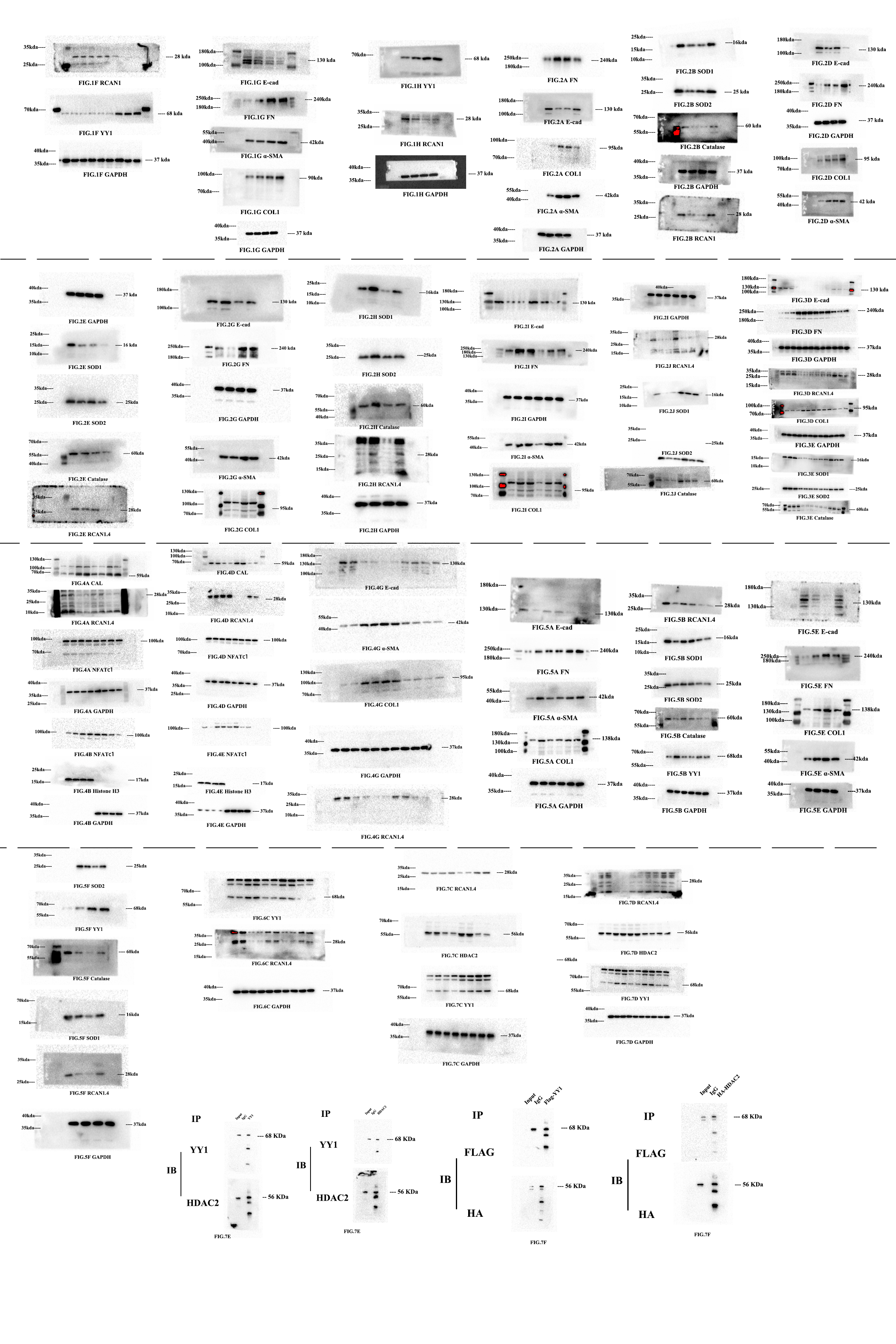

Supplement: Supplementary file 6 — Original Data File [file 41420_2023_1574_MOESM6_ESM.png]
